# Comparative study on bilateral lower extremity joint mechanics and muscle synergy patterns in Axel jumps between elite and amateur single skaters

**DOI:** 10.3389/fbioe.2025.1639807

**Published:** 2025-07-30

**Authors:** Jialiang Yu, Mingda Li, Zhiyuan Chen

**Affiliations:** ^1^ School of Sport Training, Chengdu Sport University, Chengdu, China; ^2^ Graduate School, Harbin Sport University, Harbin, China

**Keywords:** figure skating, Axel jump, joint kinematics, muscle synergy, skill level, motor control

## Abstract

**Background:**

This study aimed to reveal the differences in lower limb joint kinematic characteristics and muscle synergy patterns during the Axel jump between amateur and elite figure skaters, providing a theoretical basis for scientific training. Research on this topic, especially regarding in-depth analysis of detailed lower limb joint kinematics and muscle synergy patterns, remains insufficient.

**Methods:**

Three-dimensional motion capture systems and surface electromyography (sEMG) were used to synchronously collect kinematic and sEMG data from subjects during the approach, take-off, and flight phases of the Axel jump. OpenSim was used to process data on lower limb joint angle changes. Non-negative matrix factorization (NMF) was employed to analyze muscle synergies, muscle weighting, and activation coefficients.

**Results:**

Significant differences (P < 0.05) were found in the dynamic changes of multiple left lower limb joint angles between elite and amateur athletes during the approach-to-take-off phase. Specifically, significant differences (P < 0.05) were observed in hip flexion/extension (1%–13%), abduction/adduction (49%–53%), and external/internal rotation (1%–2%) angles at specific intervals of the movement cycle. Similarly, significant differences (P < 0.05) were found in knee flexion/extension (49%–51%), ankle dorsiflexion/plantarflexion (54%), and subtalar joint dorsiflexion/plantarflexion (21%) angles. Muscle synergy analysis revealed six synergies for the amateur group and five for the elite group. In synergy 1, related to the initial phase of the movement, the contribution weight of the left tibialis anterior was significantly higher in elite athletes (F = 15.21, P = 0.0005). In synergy 2, elite athletes activated their primary muscles during the approach-to-take-off transition phase (38%–62%), which was earlier and more concentrated than the activation in amateur athletes during the take-off-to-flight phase (59%–78%).

**Conclusion:**

The approach and take-off phases of the Axel jump are crucial for distinguishing between amateur and elite athletes. Elite athletes demonstrate more coordinated and efficient movement strategies and exhibit superior motor performance in the activation timing of key muscles. This suggests that training should focus on enhancing lower limb control capabilities and the early, efficient activation of key muscles during these phases.

## 1 Introduction

The Axel jump, first performed by Norwegian skater Axel Paulsen in 1882 and subsequently named after him, is widely recognized as the most difficult jump due to its unique forward take-off, backward landing, and additional half-rotation. Its successful execution directly impacts an athlete’s score in high-level competitions (Mazurkiewicz et al., 2018). Therefore, the Axel jump not only demands exceptional explosive power, coordination, and balance from athletes but also its mastery is often considered a hallmark of an athlete’s progression and skill level (Hirosawa, 2025). These demanding characteristics make the Axel jump a key technical challenge for both athletes and coaches. The maneuver requires athletes to master specific force generation mechanisms across its three phases: glide, take-off, and flight. Additionally, the extra half-rotation imposes greater demands on acquiring angular momentum at take-off and controlling body posture in the air (Knoll and Seidel, 2015; Helesic and Lehnert, 2024). Consequently, the Axel jump is not only a test of physical fitness but also a challenge to the precise regulation of the human neuromuscular control system. Training experience and skill level play a crucial role in shaping this regulatory ability (Rong et al., 2025): elite athletes often optimize strategies to meet competitive demands, whereas amateur athletes may demonstrate a range of suboptimal patterns, including coordination deficits or less effective force generation. Furthermore, while the stiff boot cuffs of figure skates provide ankle support, they alter the natural kinematics of the ankle and subtalar joints, compelling athletes to compensate through proximal joints (Bruening and Richards, 2006). Therefore, understanding the lower limb mechanical adjustment strategies employed by athletes of different skill levels under this constraint is crucial for developing evidence-based training programs aimed at enhancing performance and reducing injury risk.

With the development of personalized musculoskeletal modeling technology, analyzing the subtle changes in athletes’ joint angles through ice rink 3D motion capture systems combined with OpenSim musculoskeletal modeling systems helps coaches and athletes deepen their understanding of the Axel jump. For example, recent studies have successfully applied these methods to provide detailed biomechanical insights for both coaches and athletes (Shachykov et al., 2022; Bi et al., 2024). Muscle synergy refers to the functional integration of multiple muscles by the central nervous system (CNS) to form relatively fixed modules, simplifying the control of complex movements (Rana et al., 2015; Chen et al., 2023). The CNS can flexibly combine these synergistic modules to accomplish various motor tasks. Surface electromyography (sEMG) combined with algorithms such as Non-Negative Matrix Factorization (NMF) has become a common method for extracting and analyzing muscle synergy patterns (Lee and Seung, 1999; Cheung et al., 2012; Yu et al., 2024), revealing differences in how athletes of varying skill levels regulate muscle activity at the neural level. Practically, these insights provide coaches with a quantitative basis to identify the specific neuromuscular deficits of developing athletes against an elite benchmark, thereby guiding the creation of targeted training programs aimed at correcting motor pattern inefficiencies and enhancing performance.

Current biomechanical research on the Axel jump primarily focuses on kinematic parameters and sEMG analysis. In the Axel jump, the hip, knee, ankle, and subtalar joints of the lower limbs play crucial roles throughout the movement, forming a kinetic chain responsible for force generation, shock absorption, and maintaining body posture (Mazurkiewicz et al., 2018; Mazurkiewicz and Iwańska, n.d.). The kinetic chain theory posits that in multi-joint actions like jumping, force needs to be effectively transferred from proximal segments (e.g., the hip) to distal segments (e.g., the ankle) to achieve maximum movement efficacy and *vice versa*; during landing, this proximal-to-distal power transmission and reverse cushioning mechanism is essential (King et al., 1994). Existing kinematic analyses of the Axel jump have provided foundational insights into its performance. Seminal work by King et al. (1994) compared the biomechanical characteristics of single, double, and triple Axels, while more recent studies have focused on the detailed mechanics of the jump (Mazurkiewicz et al., 2018) and the specific requirements for achieving elite-level rotations (Hirosawa, 2025). These studies collectively establish that the movement patterns of the lower limb joints are key factors determining jump height, distance, and the number of rotations (Ridge et al., 2024). Among the human lower limb joints, the kinematic characteristics of the ankle joint at the moments of push-off and landing significantly affect the quality of the Axel jump, including rotation generation and landing stability (King et al., 1994; Mazurkiewicz, 2021). Research has found that higher-rotation jumps (like the triple Axel or 3A) achieve additional rotations not by significantly increasing jump height, but by reducing the moment of inertia and increasing angular velocity (Hirosawa, 2025). Elite athletes completing a 3A ensure sufficient rotations by tucking their limbs earlier and achieving faster aerial rotation speeds (up to 5 revolutions per second) (Hirosawa, 2025). In contrast, amateur athletes may experience inefficient force transfer or inadequate push-off/landing control due to suboptimal ankle strategies. Regarding muscle activity characteristics, existing studies indicate that the rectus femoris and tibialis anterior are activated first during take-off, with the rectus femoris showing the largest integrated EMG value and contribution, followed by the tibialis anterior, while the medial head of the gastrocnemius and the long head of the biceps femoris primarily serve a supporting synergistic role (Chen et al., 2023). Notably, existing research has yet to establish a systematic framework for studying the differences in lower limb kinetic parameters and muscle synergy patterns between amateur and elite athletes during the approach, take-off, and flight phases. These differential characteristics are highly likely to be core elements in breaking through technical training bottlenecks.

Numerous studies have confirmed that elite athletes exhibit significant biomechanical and motor control differences compared to amateur or novice athletes across various sports (Yoo et al., 2012; Besson et al., 2023; Marineau et al., 2024). These differences are evident in movement efficiency, force output, kinematic consistency, and neuromuscular coordination. In figure skating jumps, increases in skill level or number of rotations are typically accompanied by optimizations in kinematic parameters such as vertical take-off velocity and take-off angle (Hirosawa et al., 2023). Regarding muscle synergy patterns, research often finds that elite athletes demonstrate more refined, stable, and sometimes even fewer synergistic modules (Barnamehei et al., 2018; Matsuura et al., 2022). It is reasonable to assume that figure skaters progressing from amateur to elite levels must undergo a gradual transformation in their kinematic and neuromuscular control strategies. However, uncovering this transformation requires a comprehensive analysis combining kinematics and muscle synergies. Therefore, a comprehensive analysis combining both kinematics and muscle synergies is crucial, as it can reveal not just the quantitative differences in performance (e.g., jump height), but also the qualitative shifts in motor strategy—such as more efficient inter-joint coordination and refined synergy structures—that provide a roadmap for an athlete’s technical development.Currently, comprehensive comparative studies on the bilateral lower limb joint kinematic characteristics and muscle synergy patterns between elite and amateur figure skaters during the Axel jump are scarce. This study investigates the technical essence of the Axel jump by examining the differences in bilateral lower limb hip, knee, ankle, and subtalar joint angle changes and peak values, as well as the number of muscle synergies, muscle weightings, and activation coefficients between elite and amateur figure skaters during the three phases of the Axel jump: approach, take-off, and flight. Accordingly, this study proposes the following hypotheses: Compared to amateur athletes, elite athletes will exhibit significantly different lower limb kinematic patterns during the Axel jump, especially in the approach and take-off phases, featuring optimized joint postures conducive to force generation and rotation, as well as stronger motor control capabilities, particularly evident in the take-off leg. Compared to amateur athletes, elite athletes will demonstrate more refined muscle synergy patterns, with differences in the weighting of specific muscles within these synergies and their activation timings.

## 2 Methods

### 2.1 Participant selection

Before recruiting athletes, we used GPower 3.1.9.7 software to predict the sample size, setting the significance level (α) at 0.05 and the statistical power at 80%. The analysis indicated that a minimum sample size of 13 individuals was required. Consequently, we recruited 16 male single figure skaters, comprising 8 elite athletes and 8 amateur athletes, assigned to the elite and amateur groups, respectively. The amateur group athletes had an average age of 18.125 ± 2.41 years, body mass of 61.12 ± 4.91 kg, and height of 1.75 ± 0.04 m. The elite group athletes had an average age of 17.25 ± 2.34 years, body mass of 59.67 ± 4.58 kg, and height of 1.73 ± 0.05 m. All elite athletes were sports experts who had achieved top-six rankings in individual or overall scores in the National Games, National Championships, or National Cup competitions. The amateur group athletes had 3–5 years of figure skating training and competition experience. Neither group had a history of lower limb injuries within the year preceding the test, and all participants were aware of the test content. For the Axel jump, all athletes used their left leg as the takeoff leg and were capable of completing the experimental tasks as required. Before the experiment began, all participants were fully informed of the experimental procedures and content and signed informed consent forms. The study was conducted in accordance with all relevant ethical requirements and received formal ethical approval (approval number: 2025100).

### 2.2 Experimental procedure

The experiment was conducted on a standard figure skating rink. A twelve-camera V5 optical motion capture (OMC) system (Qualisys, Sweden) was used to record the three-dimensional kinematic data of the skaters’ movements at a sampling frequency of 100 Hz. Concurrently, a 16-channel Trigno^®^ wireless surface EMG system (Delsys, USA) was used to record surface EMG signals from major lower limb muscles at a sampling frequency of 2,000 Hz. All equipment was synchronized through the Qualisys motion capture and analysis system for coordinated signal acquisition.

During the experiment, participants wore their usual figure skating costumes and skates. They began with warm-up activities, including skating and jumping, until they reached their subjectively optimal competitive state. This also allowed them to familiarize themselves with the ice surface and the entire experimental procedure. Subsequently, reflective markers (see Table 1 for details) were placed on the participants according to the marker points of the Simbody2392 model in OpenSim. The Simbody2392 model, which serves as the foundation for lower limb motion analysis in this study, consists of 12 body segments, 23 degrees of freedom (DOFs), and 92 muscle actuators (Delp et al., 2007). Concurrently, surface electromyography (EMG) sensors were placed on the bellies of the gluteus maximus, rectus femoris, biceps femoris, tibialis anterior, gastrocnemius, and soleus muscles of both lower limbs. Before affixing the sensors, the target muscle sites were prepared by wiping the skin with alcohol to remove dry skin and grease. If there were any hairs obstructing the area, they were shaved to avoid affecting the experimental results. Finally, the surface EMG sensors were affixed along the direction of the muscle fibers using the double-sided adhesive provided with the sensors. The wireless EMG sensors were affixed directly to the prepared skin, after which participants carefully put on their tight-fitting figure skating costumes. The snug fit of the costumes also helped to secure the sensors and minimize movement artifacts during the dynamic jumps. To minimize the time gap between warm-up and testing, the instrumentation process (marker and sensor placement) was conducted efficiently by at least two experienced researchers. Crucially, after all sensors were in place, a re-warm-up period of approximately 5–10 min was provided. This allowed participants to perform several light skating movements and practice jumps to ensure they were comfortable with the equipment and had returned to their optimal competitive state immediately before data collection began.

**TABLE 1:** Specific locations of Marker points in the Gait2392_ Simbody model.

Category	Specific locations of the markers placed on the body		
Trunk	Top of the head	Left/Right Temporal Region	Sternum
	Sacrum	Left/Right Anterior Superior Iliac Spine	—
Upper Limb	Left/Right Acromion	Left/Right Biceps Brachii	Left/Right Elbow
	Left/Right Wrist Medial	Left/Right Wrist Lateral	—
Lower Limb	Left/Right Upper Thigh	Left/Right Front of Thigh	Left/Right Rear of Thigh
	Left/Right Lateral Knee	Left/Right Medial Knee	Left/Right Shank of Upper
	Left/Right Shank of Front	Left/Right Shank of Rear	Left/Right Lateral Ankle
	Left/Right Medial Ankle	Left/Right Heel	Left/Right Midfoot-Sup
	Left/Right Midfoot-Lat	Left/Right Toe Lateral	Left/Right Toe Medial
	Left/Right Toe-Tip	—	—

**FIGURE 1:**
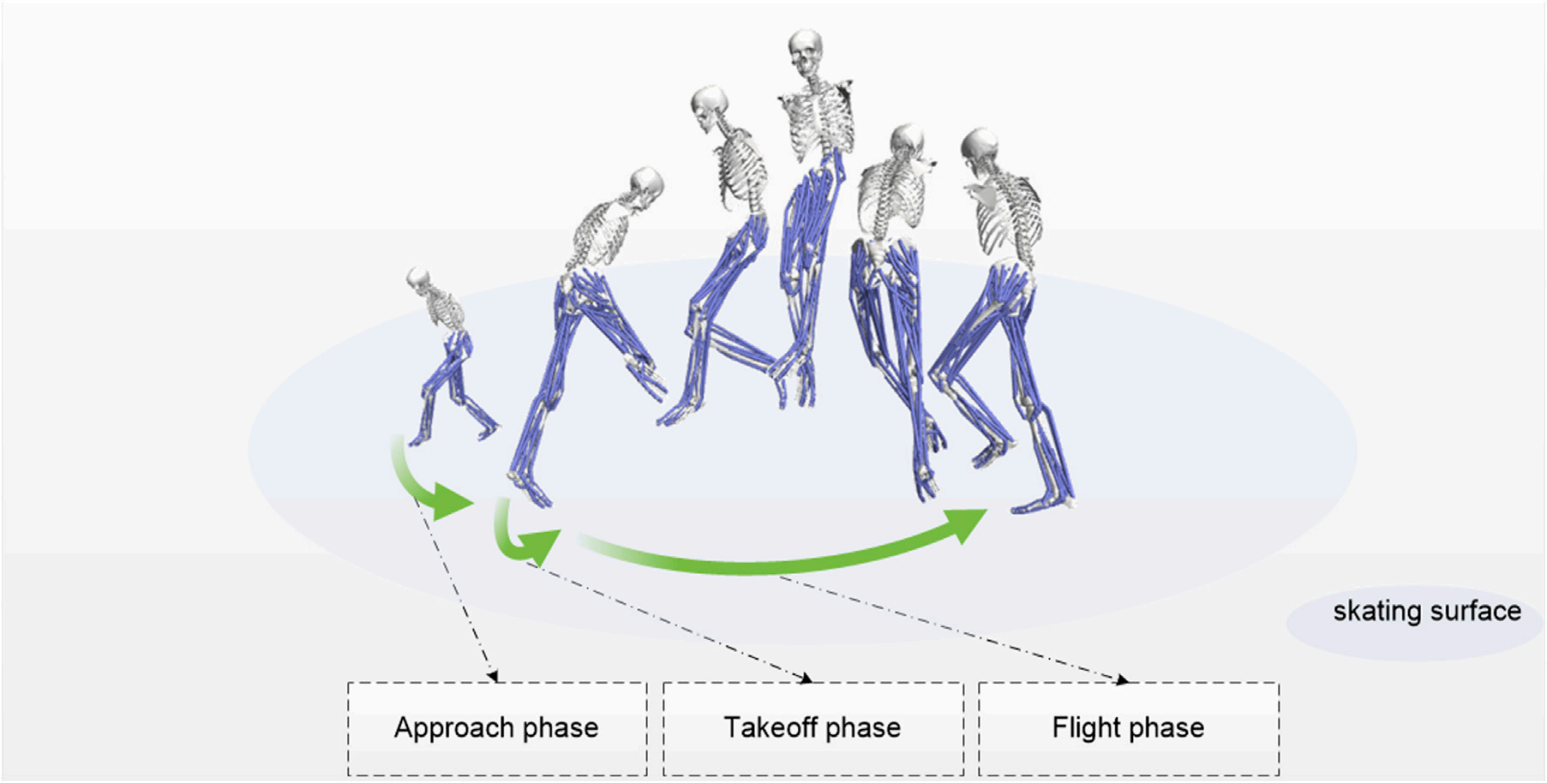
Schematic diagram of the action phase division and simulation results of the Axel jump.

After instrumentation was complete and immediately before the dynamic jump trials, a 5-s static calibration trial was recorded for each participant. During this trial, participants were instructed to stand still in the center of the capture volume in a neutral pose with their arms abducted horizontally to 90° (a T-pose). This static trial was essential for the subsequent model scaling process. After all preparations were completed, participants successively performed three Axel 2.5-revolution jumps within the camera capture range at a competitive level, with a 30-s interval between each jump. The standard procedure for the jump, which is divided into phases and illustrated schematically in Figure 1, is as follows: Participants skate counterclockwise, entering the jump with a left forward outside edge, extending the right leg backward and spreading the arms to maintain balance. They accelerate through crossover or cross-step maneuvers, skating in an arc to ensure sufficient horizontal velocity at takeoff. The left leg bends to store energy, and the takeoff is initiated by a forward outside edge push, with the body’s center of mass leaning forward. The right leg swings rapidly forward and upward to assist in generating rotational force. At the moment the left leg is fully extended, participants pre-rotate to provide initial momentum for the aerial spin, completing 2.5 revolutions (900°). Finally, the right leg (for counterclockwise skaters) lands on the back outside edge, with a slightly bent knee to cushion the impact.

### 2.3 Data processing procedure

First, the captured motion data were processed for transformation. The kinematic data were exported from the QTM software in C3D file format. Subsequently, the spatial coordinates of the data were transformed using the c3dExport.m program located in the OpenSim root directory to obtain the marker data. Next, the Signal Analysis program in MATLAB was used to apply a 10 Hz low-pass filter to both types of data to reduce noise. Following this, motion simulation was conducted. The OpenSim simulation process began with creating a personalized model for each athlete. First, using the marker data from the static calibration trial, the generic “static skeletal model” was scaled to match each participant’s individual anthropometry. This was achieved by optimizing the spatial fit between the model’s bony landmarks and the participant’s measured marker data using the least squares method in the OpenSim Scale Tool. The spatial fit between the model’s bony landmarks and the subject’s measured data was optimized using the least squares method to generate a personalized scaled model. Finally, the dynamic kinematic data were applied to the scaled model for inverse kinematics analysis to obtain kinematic parameters such as joint angles.

### 2.4 Muscle synergy extraction

EMG=W×H+ϵ (1)

This study aims to extract muscle synergy patterns, utilizing the Non-negative Matrix Factorization (NMF) algorithm (Lee and Seung, 1999), the specific mathematical formulation of is shown in Equation 1. To obtain EMG data suitable for NMF analysis, the raw signals must undergo a series of preprocessing steps: First, band-pass filtering (20–500 Hz) is applied to remove low-frequency motion artifacts, and a 60 Hz notch filter is used to eliminate power line interference. Second, the filtered signals are subjected to full-wave rectification. Finally, the signals are smoothed using a fourth-order Butterworth low-pass filter with a cutoff frequency of 6 Hz. Through these steps, the preprocessed EMG data matrix is ultimately obtained. The preprocessed EMG ( $\text{EMG}\in R^{m\times n}$ ) data are represented as an m × n matrix, where m corresponds to the number of muscle channels and n is the total number of time points. The muscle weight matrix ( $W\in R^{m\times k}$ ) quantifies the contribution of each muscle to the synergy patterns, with its elements reflecting the weight ratios of different muscles. The activation coefficient matrix ( $H\in R^{k\times n}$ ) characterizes the time-dynamic features of the synergy patterns, showing the intensity trends of each pattern over time. The residual term ( ϵ ) represents the error component of the model fit, describing the differences between the actual data and the reconstructed results from the synergy patterns. The number of synergies ( k ) for each trial was determined using a criterion based on the Variance Accounted For (VAF), which evaluates how well the reconstructed muscle synergies represent the original EMG signals.

To determine the number of synergies (k), this study selected the minimum number of synergies required to account for at least 98% of the variance in the original EMG signal (VAF ≥ 98%) as the optimal dimensionality (Allami Sanjani et al., 2023). Adopting this high-fidelity threshold aims to ensure the functional adequacy of the extracted synergy modules, sufficient to capture the subtle but critical features of muscle activation patterns necessary for complex technical movements (Sabzevari et al., 2017; Fukunishi et al., 2024). Additionally, we used K-means clustering to group individual synergy patterns. The formula for calculating VAF is as follows:

$$\text{VAF}=1-\frac{∥\text{EMG}-\text{WH}{∥}_{F}^{2}}{∥\text{EMG}{∥}_{F}^{2}}$$ (2)

where RSS (Residual Sum of Squares) is calculated as the squared Frobenius norm of the error matrix ( $∥\text{EMG}-\text{WH}{∥}_{F}^{2}$ ), and TSS (Total Sum of Squares) is the squared Frobenius norm of the original data matrix ( $∥\text{EMG}{∥}_{F}^{2}$ ). The residual sum of squares (RSS) and the total sum of squares (TSS) were calculated according to Equation 2. A larger VAF indicates a more accurate representation of the original data by the decomposition model. To further ensure the reliability of the extracted synergy vectors (W), a consistency analysis was performed across multiple NMF decompositions for each participant. The stability of the resulting synergy vectors was evaluated using the Intra-Class Correlation coefficient (ICC). Following established guidelines, ICC values greater than 0.75 were considered to represent excellent reliability. Any muscle channels demonstrating poor stability (ICC < 0.5) were considered for exclusion from the final synergy analysis (Koo and Li, 2016).

### 2.5 Phase division of movement and key observation indicators

In this study, the Axel jump is divided into three phases based on whether the skater’s blades are in contact with the ice and the changes in the center of mass, as shown in Figure 1. During the glide phase, both of the skater’s feet remain in contact with the ice. The skater adjusts the gliding speed by pushing off the ice to prepare for takeoff. For the purpose of this study, this phase was defined as ending when the center of mass reaches its lowest point, as our observational data showed this event consistently marked the transition from the preparatory glide to the upward propulsive action. The takeoff phase begins when the takeoff foot starts to push off the ice and ends when the blade fully leaves the ice. During this phase, the skater rapidly extends the legs, converting the horizontal gliding speed into upward force and rotational momentum. This allows the body to gain upward velocity and an initial rotational tendency. The airborne phase begins when the blade leaves the ice and ends just before landing. During this phase, the skater completes the rotation in the air while adjusting the body posture to prepare for a smooth landing.

The main research indicators include the following:

(1) Changes in the joint angle curves of the athletes’ bilateral lower limbs:

In the joint angle curves of the lower limbs, the symbols “+” and “−” respectively indicate: Hip flexion (+) and extension (−) angles; Hip adduction (+) and abduction (−) angles; Hip medial rotation (+) and lateral rotation (−) angles; Knee flexion (−) and extension (+) angles; Ankle dorsiflexion (+) and plantarflexion (−); Subtalar inversion (−) and eversion (+) angles.

(2) Peak joint angles:

The peak joint angles are already marked with their respective states in the tables. Therefore, no symbols are used to indicate changes in the data, and all values are positive.

(3) Number of lower limb muscle synergies:

Muscle participation weights and activation coefficients in muscle synergies. To identify the principal muscles contributing to each synergy, a weighting threshold was applied. Consistent with conventions used in prior synergy research (Kubota et al., 2021; Zhang et al., 2024), muscles with a weight ≥ 0.3 in a given synergy vector were considered to be significant contributors to that synergy pattern. In the activation coefficient matrix, the normalized absolute value ≥0.3 is set as the activation threshold to determine the temporal activation state of the corresponding synergy pattern. It should be noted that “non-activated muscles” do not imply complete physiological inactivity. Instead, they indicate that their activation weights in the synergy pattern are below 0.3, reflecting differences in muscle participation rather than the presence or absence of physiological activity.

### 2.6 Statistical analysis

In this study, statistical analyses were conducted using MATLAB 2023a and IBM SPSS Statistics 24 software. For the joint angle curve data of the bilateral lower limbs, all data were first time-normalized using MATLAB software. The original data were interpolated to 101 data points, with 0% representing the start of the movement and 100% representing the end of the movement. Subsequently, based on the time-normalized data, a one-dimensional statistical parametric mapping (SPM1d) F-test was employed for analysis. A two-tailed test was used with a significance level set at 0.05 to compare differences between the two groups’ data throughout the movement cycle (Pataky et al., 2016). For the peak joint angle data and muscle weight data in muscle synergies, results are presented as mean ± standard deviation. One-way analysis of variance (ANOVA) was used to explore significant differences between groups. Prior to ANOVA, data were tested for normal distribution and homogeneity of variance. When variances were homogeneous, the Bonferroni method was used for multiple comparisons correction; when variances were heterogeneous, Tamhane’s T2 method was selected. In both cases, the significance level was set at 0.05. Additionally, the effect size η2was calculated to comprehensively assess the practical significance of the study results.

## 3 Results

### 3.1 Kinematic characteristics

**FIGURE 2:**
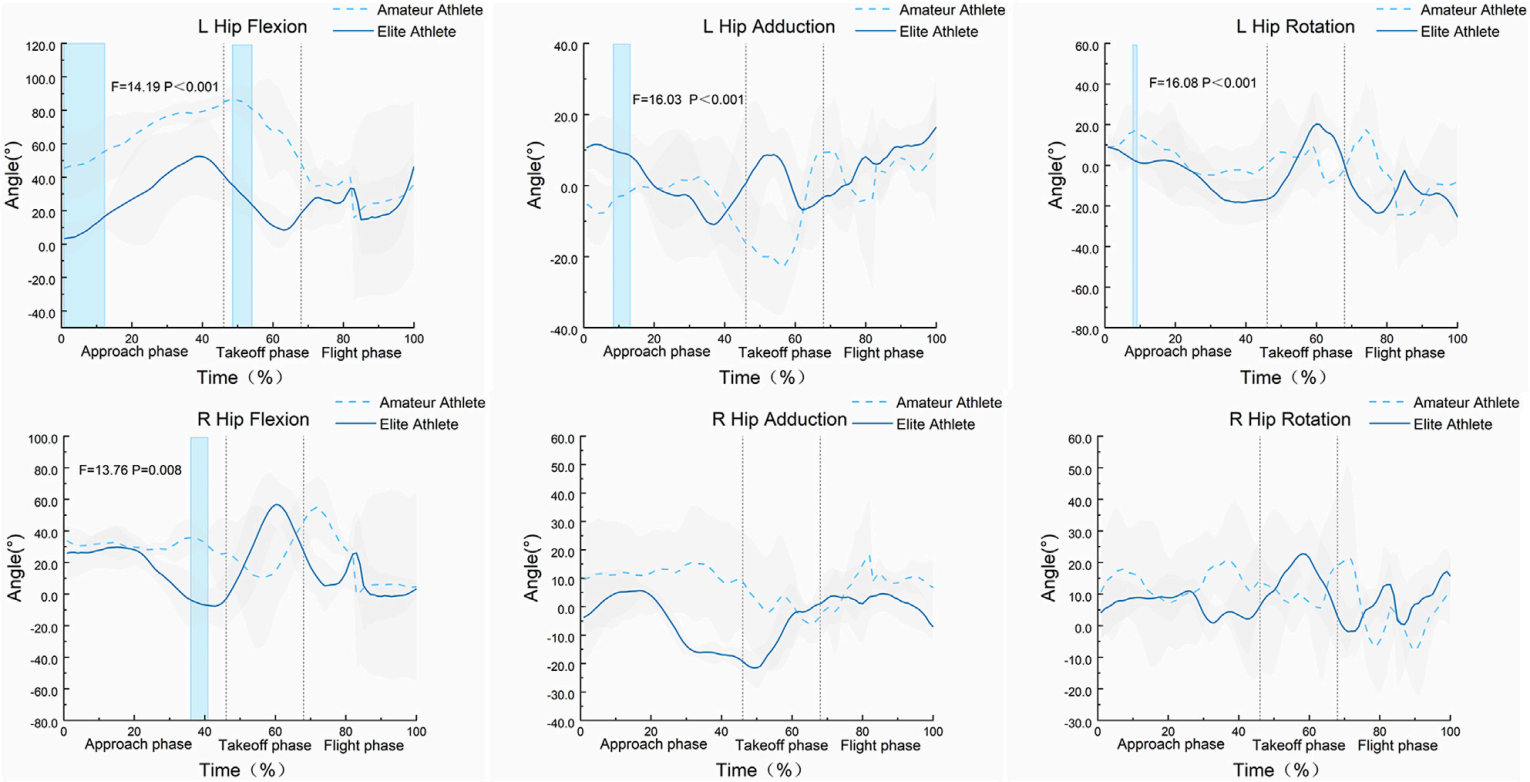
Bilateral hip joint angles of the Axel jump phases and SPM1d{F} statistical analysis for amateur and elite athletes (n = 16). Figure legend: Areas with a light blue background and dark border indicate statistically significant differences observed between the two groups during that time period.

The SPM1d{F} statistical results shown in Figure 2 indicate that there are significant differences in the hip joint angle variation curves of the Axel jump between amateur and elite athletes during specific phases of the entire movement. Specifically, significant differences in the flexion-extension angles of the left hip joint were observed during the time intervals of 1%–13% and 49%–53% (α = 0.05, F = 14.19). Significant differences in the abduction-adduction angles of the left hip joint were found during the time interval of 9%–13% (α = 0.05, F = 16.03). Significant differences in the external-internal rotation angles of the left hip joint were detected during the time interval of 1%–2% (α = 0.05, F = 16.08). Additionally, significant differences in the flexion-extension angles of the right hip joint were observed during the time interval of 38%–41% (α = 0.05, F = 13.76). However, the SPM1d{F} statistical results for the abduction-adduction and external-internal rotation angles of the right hip joint did not show significant differences.

**FIGURE 3:**
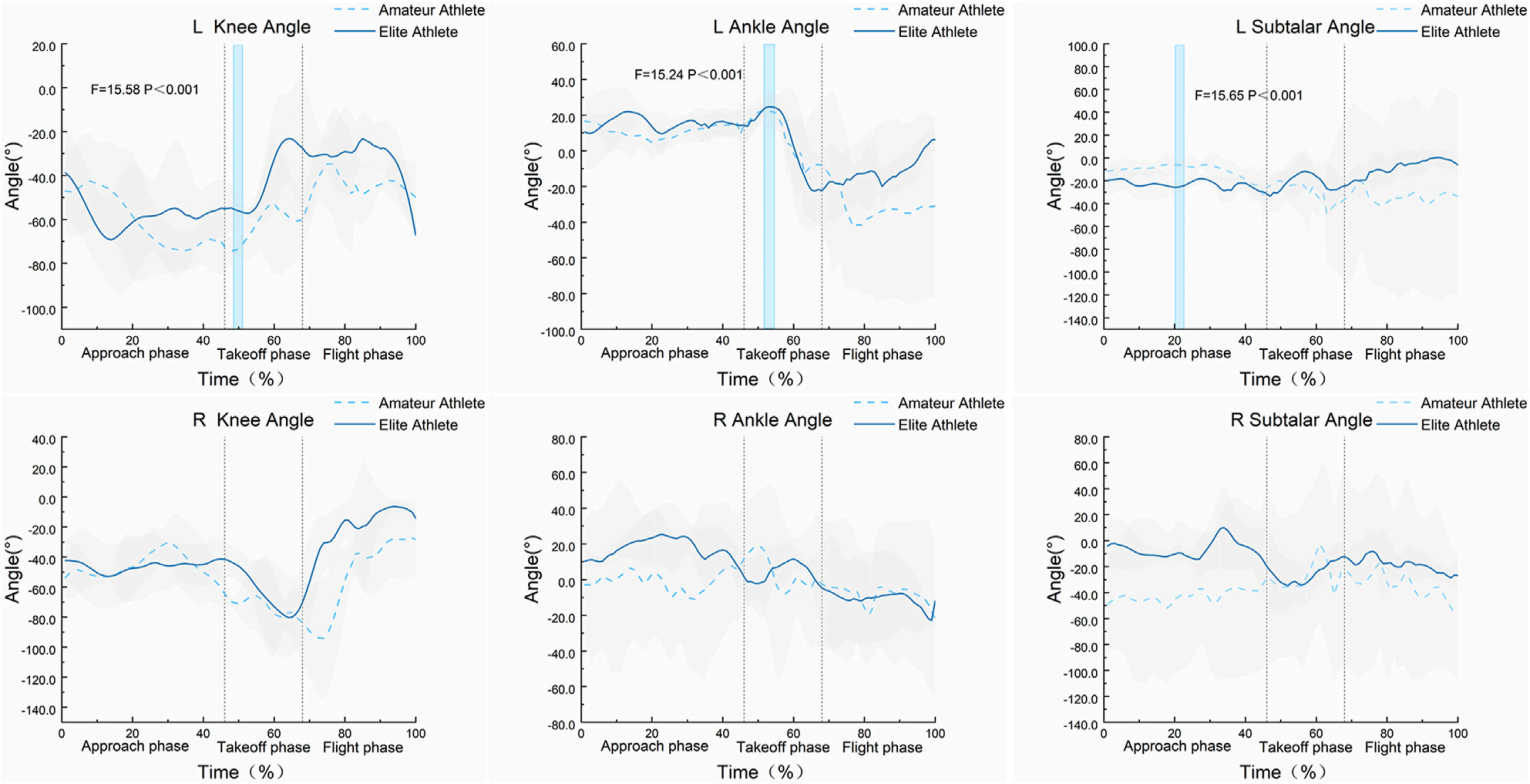
Bilateral knee, ankle, and subtalar joint angles of the Axel jump phases and SPM1d{F} statistical analysis for amateur and elite athletes (n = 16). Figure legend: Areas with a light blue background and dark border indicate statistically significant differences observed between the two groups during that time period.

The SPM1d{F} statistical results shown in Figure 3 indicate that during the entire movement process, there are significant differences in the angle variation curves of the left knee, ankle, and subtalar joints during specific phases of the Axel jump between amateur and elite athletes. Specifically, significant differences in the flexion-extension angles of the left knee joint were observed during the time interval of 49%–51% (α = 0.05, F = 15.58). Significant differences in the flexion-extension angles of the left ankle joint were found at the time point of 54% (α = 0.05, F = 15.24). Significant differences in the flexion-extension angles of the left subtalar joint were detected at the time point of 21% (α = 0.05, F = 15.65). In contrast, the SPM1d{F} statistical results for the angle variation curves of the right knee, ankle, and subtalar joints did not show significant differences.

**FIGURE 4:**
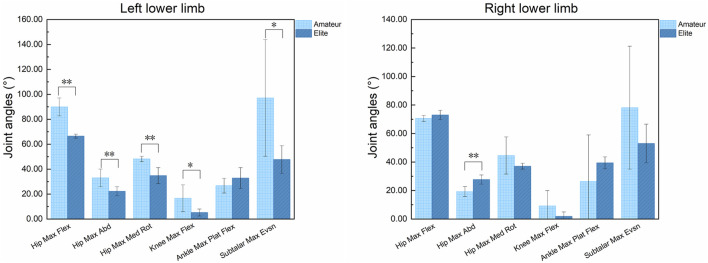
Comparison of peak bilateral lower limb joint angles during the Axel jump between elite and amateur athletes. Figure Caption:Asterisks indicate a statistically significant difference between the amateur and elite groups (p < 0.05, *p < 0.01).

As shown in Table 2 and Figure 4, there are differences in the joint angle characteristics of the left lower limb during the entire Axel jump between amateur and elite athletes. Specifically, the maximum flexion angle of the left hip joint in elite athletes is significantly smaller than that in amateur athletes (P < 0.001); the maximum abduction angle of the left hip joint in elite athletes is significantly smaller than that in amateur athletes (P < 0.05); the maximum internal rotation angle of the left hip joint in elite athletes is significantly smaller than that in amateur athletes (P < 0.001); the maximum flexion angle of the left knee joint in elite athletes is significantly smaller than that in amateur athletes (P < 0.05); the maximum eversion angle of the left subtalar joint in elite athletes is significantly smaller than that in amateur athletes (P < 0.05). In addition, the maximum abduction angle of the right hip joint in elite athletes is significantly larger than that in amateur athletes (P < 0.05). No significant differences were found in the other lower limb joint angle characteristics.

**TABLE 2:** Analysis of the differences in joint angle characteristics of bilateral lower limbs during Axel jumps between amateur and elite athletes (n = 16).

Variable	Amateur athletes n = 8		Elite athletes n = 8		F	P	η2
	Mean SD		Mean SD				
L Hip Max Flex Ang	89.93	7.23	66.45	1.60	62.42	**<0.01**	**0.89**
L Hip Max Abd Ang	32.98	7.09	22.26	3.63	10.20	**0.01**	**0.56**
L Hip Max Med Rot Ang	48.17	2.14	34.78	6.43	15.59	**<0.01**	**0.66**
L Knee Max Flex Ang	16.63	10.80	5.20	2.82	6.44	**0.04**	**0.45**
L Ankle Max Plat Flex Ang	26.72	5.87	32.87	8.35	1.61	0.24	**0.17**
L Subtalar Max Evsn Ang	97.16	46.78	47.74	11.08	6.53	**0.03**	**0.45**
R Hip Max Flex Ang	70.61	2.09	73.00	3.24	1.67	0.23	**0.17**
R Hip Max Abd Ang	19.23	3.52	27.70	3.22	15.45	**<0.01**	**0.66**
R Hip Max Med Rot Ang	44.60	13.04	37.06	2.04	2.06	0.19	**0.20**
R Knee Max Flex Ang	9.19	10.73	1.88	3.06	2.61	0.15	**0.25**
R Ankle Max Plat Flex Ang	26.33	32.71	39.44	4.19	1.00	0.35	0.11
R Subtalar Max Evsn Ang	78.17	43.16	53.00	13.56	1.87	0.21	**0.19**

Table Notes: The numerical definitions of the effect size indicator η^2^ are as follows: a value of 0.01 indicates a small effect, 0.06 indicates a medium effect, and 0.14 indicates a large effect.

### 3.2 Muscle synergy characteristics

**FIGURE 5:**
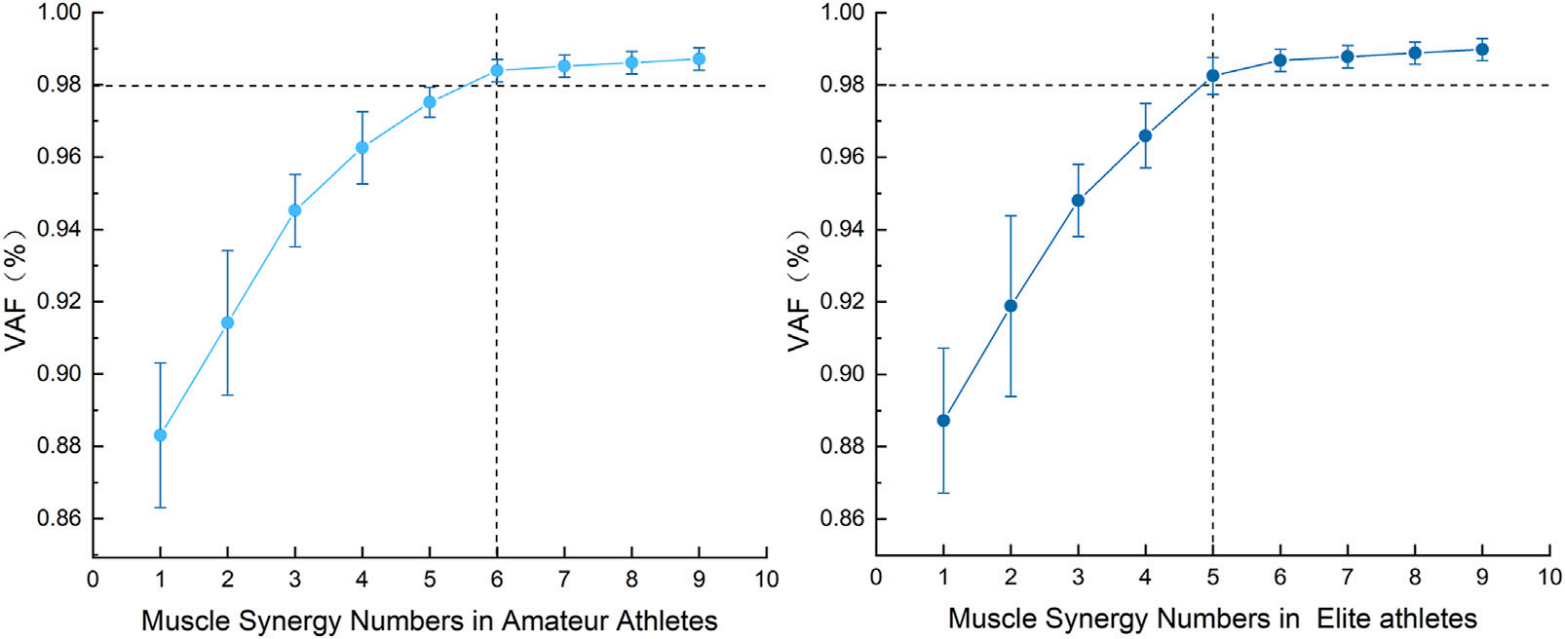
Comparison of the number of muscle synergies and VAF (Variance Accounted For) in bilateral lower limbs during Axel jumps between amateur and elite athletes.

As shown in Figure 5, the study found that during the Axel jump, the muscles of the bilateral lower limbs of amateur athletes, analyzed using Non-negative Matrix Factorization (NMF), achieved a Variance Accounted For (VAF) of 98.25% when the number of muscle synergies was 6, resulting in the extraction of 6 synergies. In contrast, for elite athletes, the muscles of the bilateral lower limbs, analyzed using the same method, achieved a VAF of 98.39% when the number of synergies was 5, resulting in the extraction of 5 synergies.

**FIGURE 6:**
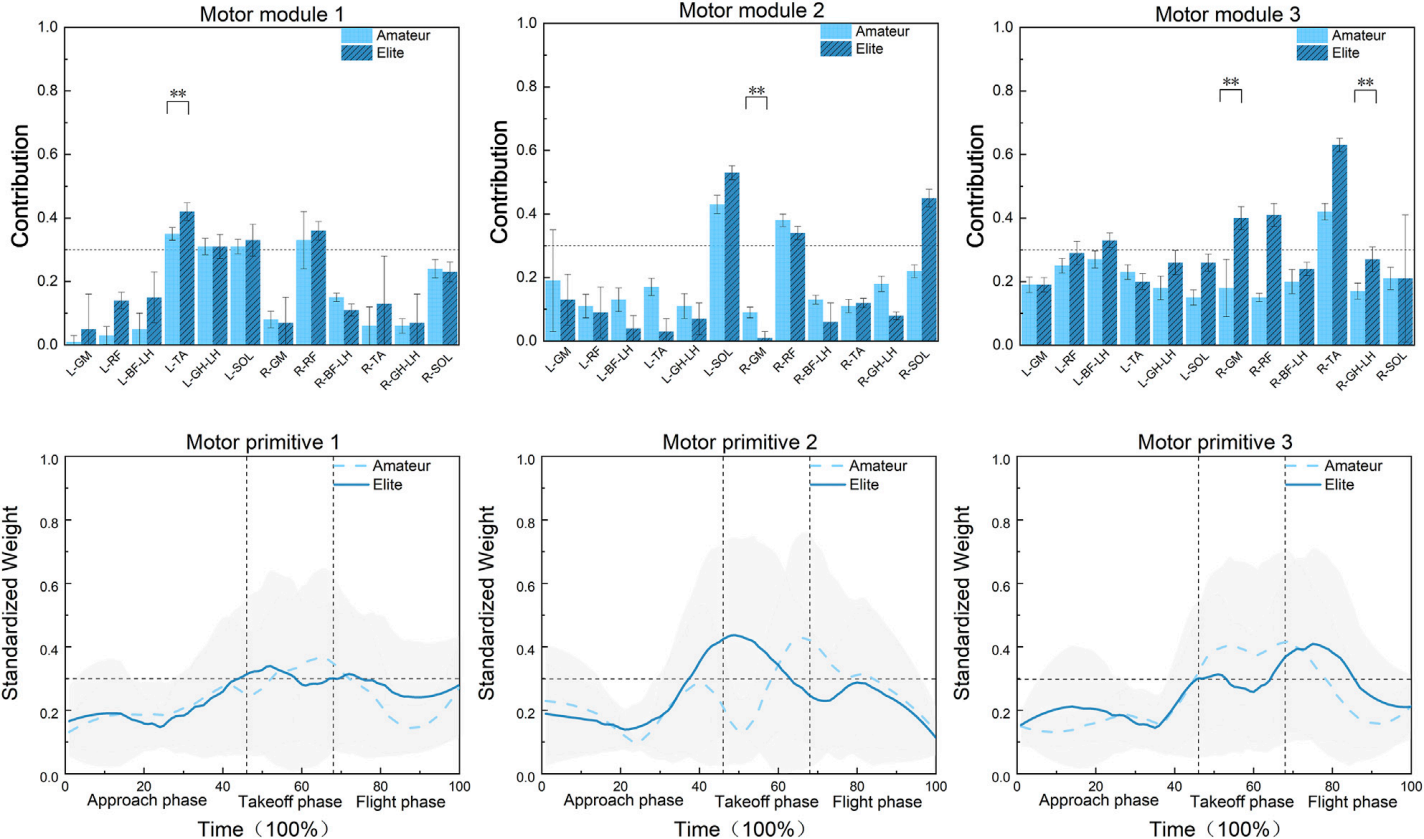
Modular contributions and weights of muscle synergies 1-3 in the bilateral lower limbs during Axel jumps between amateur and elite athletes.

As shown in Figure 6, in Synergy 1, the primary muscles involved in the Axel jump for both groups of athletes, in order of contribution size, are the left tibialis anterior, right rectus femoris, left soleus, and lateral head of the left gastrocnemius. Among them, the contribution weight of the left tibialis anterior in elite athletes is significantly higher than that in amateur athletes (F = 15.21, P = 0.0005).

In amateur athletes, the activation curves of these primary muscles are activated during 53%–70% of the movement cycle, mainly concentrated in the take-off phase. In contrast, in elite athletes, the activation curves of these muscles are activated during 42%–58% and 68%–75% of the movement cycle, also mainly concentrated in the take-off phase. In Synergy 2, the primary muscles involved in the Axel jump for amateur athletes, in order of contribution size, are the left soleus and right rectus femoris. For elite athletes, the primary muscles involved, in order of contribution size, are the left soleus, right soleus, and right rectus femoris. There were no significant differences in the contributions of the primary muscles between the two groups. However, among the non-primary muscles, the contribution of the right gluteus maximus was significantly different between the groups, with the amateur group having a higher contribution than the elite group (F = 13.43, P < 0.001).

In amateur athletes, the activation curves of these primary muscles are activated during 59%–78% of the movement cycle, mainly distributed in the interval between the take-off phase and the airborne phase. In elite athletes, the activation curves of these primary muscles are activated during 38%–62% of the movement cycle, mainly distributed in the interval between the glide phase and the take-off phase.

In Synergy 3, the primary muscle involved in the Axel jump for amateur athletes is the right tibialis anterior. For elite athletes, the primary muscles involved, in order of contribution size, are the right tibialis anterior, right rectus femoris, right gluteus maximus, and long head of the left biceps femoris. Among them, the contribution of the right gluteus maximus was significantly different between the two groups, with the amateur group having a lower contribution than the elite group (F = 9.71, P = 0.004). Additionally, among the non-primary muscles, the contribution of the lateral head of the right gastrocnemius was also significantly different, with the amateur group having a lower contribution than the elite group (F = 8.2, P = 0.007).

In amateur athletes, the activation curves of these primary muscles are activated during 46%–85% of the movement cycle, mainly distributed in the interval between the take-off phase and the first half of the airborne phase. In elite athletes, the activation curves of these primary muscles are activated during 65%–85% of the movement cycle, mainly distributed in the interval between the second half of the take-off phase and the first half of the airborne phase.

**FIGURE 7:**
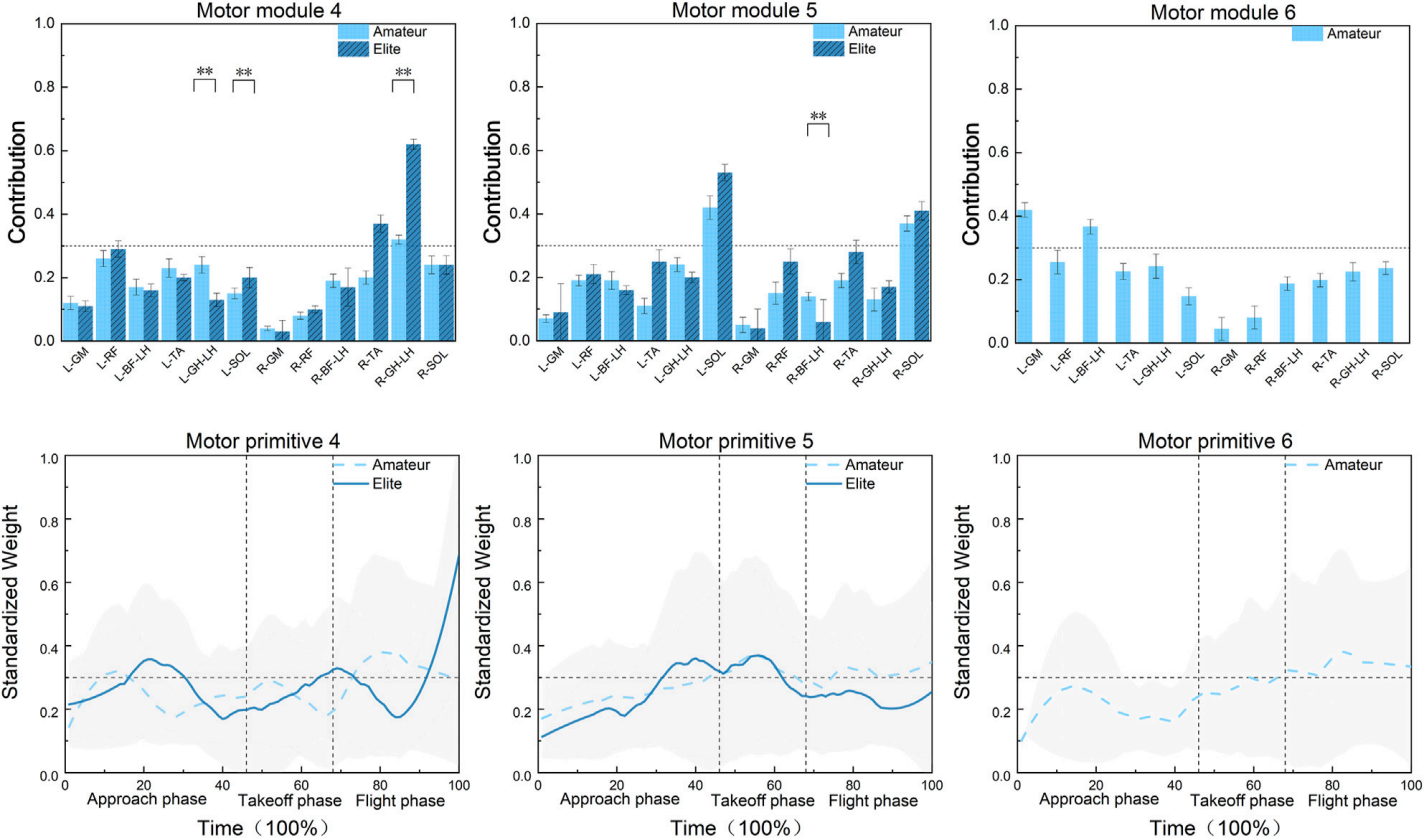
Modular contributions and weights of muscle synergies 4-6 in the bilateral lower limbs during Axel jumps between amateur and elite athletes.

Shown in Figure 7, in Synergy 4, the primary muscle involved in the Axel jump for amateur athletes is the lateral head of the right gastrocnemius. For elite athletes, the primary muscles involved, in order of contribution size, are the lateral head of the right gastrocnemius and the right tibialis anterior. The contribution of the right tibialis anterior is significantly different between the two groups, with the amateur group having a lower contribution than the elite group (F = 4.66, P = 0.039). Among the non-primary muscles, the contributions of the lateral head of the left gastrocnemius and the left soleus are also significantly different, with the amateur group having higher contributions than the elite group (F = 4.88, P = 0.035 for the lateral head of the left gastrocnemius; F = 6.43, P = 0.017 for the left soleus). In amateur athletes, the activation curves of these primary muscles are activated during 9%–13% and 73%–100% of the movement cycle, mainly distributed in the glide phase and airborne phase. In elite athletes, the activation curves of these primary muscles are activated during 16%–30% and 90%–100% of the movement cycle, also mainly distributed in the glide phase and airborne phase.

In Synergy 5, the primary muscles involved in the Axel jump for both groups of athletes, in order of contribution size, are the left soleus and right soleus. There are no significant differences in the contributions of the primary muscles between the two groups. However, among the non-primary muscles, the contribution of the long head of the right biceps femoris is significantly different, with the amateur group having a higher contribution than the elite group (F = 10.52, P = 0.0029). In amateur athletes, the activation curves of these primary muscles are activated during 46%–68% and 75%–100% of the movement cycle, mainly distributed in the take-off phase and airborne phase. In elite athletes, the activation curves of these primary muscles are activated during 30%–60% of the movement cycle, mainly distributed in the second half of the glide phase and the first half of the take-off phase.

In Synergy 6, the primary muscles involved in the Axel jump for amateur athletes are the left gluteus maximus and the long head of the left biceps femoris, with other muscles being non-primary contributors. The activation curves of these primary muscles are activated during 68%–100% of the movement cycle, mainly distributed in the airborne phase.

## 4 Discussion

The study aims to examine the differences in bilateral lower limb joint kinematics and muscle synergy patterns between elite and amateur figure skaters during the Axel jump. It involves a detailed comparison of joint kinematics, muscle activity organization, coordination, and neuromuscular control mechanisms across multiple phases of the jump, including the approach, take-off, and flight. The objective is to identify the differences in neuromuscular control strategies and biomechanical optimization mechanisms between elite and amateur athletes during the performance of high-difficulty jumps like the Axel.

### 4.1 Differences in Axel jump kinematic characteristics between elite and amateur figure skaters

Previous research indicates that completing an Axel jump requires precise control and effective preparation during the glide and take-off phases (Mazurkiewicz et al., 2018), findings that align with the results and conclusions of this study. The glide phase, which is preparatory for the Axel jump, directly impacts the subsequent take-off. According to the SPM1d results of this study, elite athletes exhibited significant differences in left hip flexion/extension, abduction/adduction, and medial/lateral rotation during the glide phase (1%–13%, 9%–13%, and 8%–9% of the movement cycle, respectively) compared to amateur athletes. This suggests that the kinematic differences in the left hip joint between the two groups are primarily concentrated in the glide phase. It also implies that from the initial preparation to the force generation stage, the control pattern of hip flexion/extension in the support leg fundamentally differs between elite and amateur athletes, establishing a technical advantage from the outset. Similar findings were reported by King et al., who emphasized that the take-off preparation and take-off phases are key to differentiating Axel jumps of varying skill levels (King et al., 1994). In this study, elite athletes had significantly smaller hip flexion angles during the take-off phase compared to amateur athletes, with SPM1d and discrete variable results showing similar trends. As a crucial joint in the lower limbs, the hip’s range of motion during the glide phase directly influences an athlete’s ability to fully extend and contract muscle groups during take-off, thereby generating greater vertical velocity and force, which are critical determinants for achieving a higher number of rotations and thus a successful jump (King et al., 1994; Mazurkiewicz et al., 2018). The smaller left hip flexion angle in elite athletes during the glide phase may allow for more precise control of hip flexion and extension timing, preparing for the subsequent ice push-off and free leg swing (Secomb et al., 2021). Related studies often highlight the importance of center of mass stability and trajectory during the glide for take-off (Korsch et al., 2023). A smaller hip flexion angle during gliding also allows the body’s center of mass to be closer to the ice surface, reducing air resistance and improving gliding efficiency (Secomb et al., 2021; Wei et al., 2023). The glide phase of the Axel jump involves crossover steps, requiring the hip to rapidly transition from extension to abduction during the push-off to generate sufficient angular momentum (Hirosawa, 2025). A smaller flexion angle helps athletes quickly transition to the push-off posture, reducing time lost in joint angle adjustment. The choice of a smaller hip flexion/extension angle by elite athletes is a result of a comprehensive trade-off between speed, power. Furthermore, the ability to maintain this smaller angle under the high forces of the glide phase is indicative of superior hip strength and static stability. This aligns with the findings of Secomb et al. (2021), who highlighted the importance of joint angle-specific strength for skating performance. This study also found significant differences in the left hip abduction/adduction angles between elite and amateur athletes during the 9%–13% interval of the movement. Hip abductor muscles are vital for single-leg jump stability and frontal plane knee control (Cronin et al., 2016; Miyamoto et al., 2023). Hip movement in the frontal plane is crucial for maintaining body balance, controlling the skate blade’s attitude, and initiating rotation (Gribble and Hertel, 2004). This indicates that elite athletes exhibit more optimized abduction/adduction angle control to ensure stable support of the push-off leg on the ice and to prepare for the transfer of the center of mass and the initiation of rotation. This study also found a significant difference in the subtalar joint eversion angle at the 21% time interval. While the stiff skating boot significantly constrains the range of motion at this joint, our findings suggest that skill-based differences in control still exist within this limited range. Therefore, we interpret the smaller maximum eversion angle in elite athletes not as a large, free movement, but as evidence that they have developed a more refined strategy to maximize medial-lateral stability on the blade edge. This superior stability might be achieved through more effective isometric contractions of the ankle musculature, preventing undesirable micro-movements within the boot and creating a more rigid foot-boot unit. From this, it can be inferred that elite athletes may place greater emphasis on the preparatory nature and precision of movement during the glide phase. By making subtle adjustments to the hip joint and controlling the subtalar joint eversion angle, they lay the foundation for the subsequent explosive take-off. For amateur athletes, training should focus on controlling the hip and subtalar joints during the glide phase. Training should not only aim to improve gliding speed but also emphasize the stability of the gliding posture and the preparatory stance of the propulsive leg (Hendricks et al., 2024).

The take-off phase is the core segment where energy accumulated during the glide phase is converted into the vertical and rotational momentum required for flight (Shi et al., n.d.). During this phase, significant multi-joint and multi-level kinematic differences were observed between elite and amateur athletes. Elite athletes demonstrated a significantly smaller left hip maximum flexion angle. This aligns with the previously discussed concept that “elite athletes build a technical advantage from the very beginning.” Although reduced hip joint range of motion might lead to compensatory movements, in this context, it appears to be an optimized strategy rather than a functional limitation. Elite athletes also exhibited a significantly smaller left hip maximum abduction angle, potentially indicating a greater focus on direct upward force generation to maintain a more compact axis of rotation during the flight phase. Furthermore, elite athletes had a significantly smaller left hip maximum internal rotation angle, which is a key finding for understanding the rotation mechanism. This may imply that elite athletes do not need to rely on extreme joint angles to complete the necessary pre-rotation, as excessive internal rotation could lead to instability (Li et al., 2019).

In the 49%–51% time interval (i.e., the take-off phase), a significant difference was observed in the flexion/extension angle of the left leg knee joint. More importantly, data from Table 2 indicate that the maximum flexion angle of the left knee joint in elite athletes was significantly smaller than that in amateur athletes (P < 0.05). This finding is consistent with the characteristics of high-level athletes in some jumping sports, who do not pursue maximum joint flexion but rather achieve an “optimal” flexion angle (Di Domenico et al., 2024). This strategy of a smaller maximum knee flexion angle reveals a more “tuned” and efficient stretch-shortening cycle (SSC) mechanism in the use of the left leg knee joint by elite athletes (Flanagan and Comyns, 2008). That is, they can achieve optimal muscle pre-stretching and elastic energy storage with smaller joint displacements. This smaller range of flexion allows the knee extensor muscles to operate closer to their optimal force-length and force-velocity curve ranges, thereby promoting rapid and powerful extension (Graham-Smith and Lees, 2005). In contrast, amateur athletes, possibly due to greater knee flexion, may experience a slower take-off process, generate less force, and dissipate more energy rather than effectively returning it elastically.

In the 54% time interval (i.e., the preparatory stage of take-off), a significant difference was observed in the flexion/extension angle of the left ankle joint. Some studies have pointed out that in the preparatory stage of the Axel jump take-off, reducing the ankle joint angle (i.e., greater dorsiflexion followed by rapid plantarflexion) is crucial for completing the rotation, as this increases pressure on the skate blade, thereby reducing horizontal velocity and increasing the vertical take-off angle (Mazurkiewicz et al., 2018). During the flight phase, the results of this study show smaller differences in bilateral lower limb joint angles between the two groups. This is consistent with the research conclusion that the glide and take-off phases are the important action phases of the Axel jump.

Thus, the dynamic joint angle differences between amateur and elite athletes during the Axel jump are primarily focused on the multi-joint coordination of the left lower limb, especially the adjustments of the hip joint during the glide phase and the fine-tuning and control strategies of multiple joint angles in the left lower limb during the take-off phase. These findings provide key biomechanical optimization points for the technical training of both amateur and elite athletes, helping the former to bridge foundational skill gaps and the latter to identify and correct subtle inefficiencies in their technique.

### 4.2 Differences in Axel jump muscle synergy control patterns between elite and amateur figure skaters

Synthesizing the results from all synergies, we found that elite figure skaters employ a more economical, refined, and efficient neuromuscular control strategy when performing the Axel jump. Specifically, after analysis using the NMF decomposition algorithm, elite athletes exhibited five synergies, whereas amateur athletes had six. Although elite athletes had fewer muscle synergies, both groups achieved a similarly high level in explaining the total variance accounted for (VAF) of muscle activity. This phenomenon is generally considered a hallmark of more precise and efficient human motor control (Safavynia et al., 2011). Within the five similar synergies shared by both groups of athletes, we revealed differences in neuromuscular control strategies between elite and amateur athletes.

In synergy 1, the primarily involved muscle groups are mainly activated during the take-off phase. Among these, the contribution weighting of the left tibialis anterior (LTA) in elite athletes was significantly higher than in amateur athletes. The left tibialis anterior plays a role in ankle dorsiflexion, controlling skate blade posture during gliding, and maintaining ankle joint stability. This indicates that elite athletes can utilize the left tibialis anterior to precisely regulate ankle mechanics and skate blade posture during take-off, which helps in achieving an optimal take-off angle. Furthermore, studies have shown that fine control of the ankle joint, involving initial dorsiflexion followed by plantarflexion, is crucial for the subsequent take-off rotation. This reflects elite athletes’ more advanced ability of critical movement junctures and more optimized neural control.

In synergy 2, the primary participating muscles were the soleus and rectus femoris, mainly involved in the transition phase from glide to take-off. The soleus primarily contributes to ankle plantarflexion, providing powerful impetus for the ice push-off. The rectus femoris, as a biarticular muscle, participates in knee extension and hip flexion and is crucial for force transmission in explosive jumping movements (Babic and Lenarcic, 2007). The activation window for elite athletes was in the 38%–62% interval of the movement, encompassing the glide to take-off phase, indicating that they begin accumulating and preparing force from the glide phase. However, amateur athletes exhibited later activation. While the principal muscles (weights ≥ 0.3) define a synergy’s main function, significant differences in the weighting of non-primary muscles can be highly informative, often highlighting compensatory or less refined motor strategies. Amateur athletes showed a significantly higher contribution of the non-primary participating muscle RGM (right gluteus maximus) in this synergy, potentially recruiting other muscle groups like the gluteus maximus more to assist in force generation as a compensatory mechanism (Li et al., 2025).

In synergy 3, the primary participating muscle groups for the elite group were the right tibialis anterior, right rectus femoris, right gluteus maximus, and left biceps femoris long head. The muscle composition was more complex, and activation was more concentrated, crucial for increasing height and rotation. In contrast, the primary participating muscle group for the amateur group was the right tibialis anterior. The difference between the two groups was significant. Elite athletes demonstrated a highly coordinated, explosive force generation pattern involving control over multiple large muscle groups in synergy 3. The combined high involvement of RRF (right rectus femoris, knee extension), RGM (right gluteus maximus, hip extension), and RGCAL (right gastrocnemius lateral head, plantarflexion) constitutes a typical “triple extension” power chain, which is key to generating large ground reaction forces to achieve flight height (Babic and Lenarcic, 2007).

Synergy 4 was activated during both the gliding and flight/landing preparation phases of the Axel jump. Amateur athletes primarily relied on the right gastrocnemius lateral head (RGCAL). Elite athletes, however, simultaneously activated both the right gastrocnemius lateral head (RGCAL) and the right tibialis anterior (RTA), with the contribution of RTA being significantly higher than in the amateur group. The simultaneous activation (co-contraction) of RGCAL and RTA in elite athletes may help increase ankle joint stiffness, providing a more stable support platform for the subsequent ice push-off and take-off, and enabling fine control of the skate blade posture (Mazurkiewicz et al., 2018).

In synergy 5, both groups showed the left soleus (LSOL) and right soleus (RSOL) as the primary contributing muscles, indicating the core role of bilateral ankle plantar flexor groups in this synergy, likely serving primarily for vertical explosive force generation (Brockett and Chapman, 2016). The significantly higher non-primary contribution of the right biceps femoris long head (RBFLH, a hip extensor and knee flexor (Padulo et al., 2013)) in amateur athletes in this synergy might indicate unnecessary co-contraction. Elite athletes can typically activate target muscle groups more precisely, reducing non-functional or compensatory muscle co-activation, thereby improving movement energy efficiency and control precision (Yaserifar et al., 2021). The higher RBFLH activation in amateur athletes might be due to an inability to effectively separate hip-knee control from ankle explosive force when attempting to enhance jump power or maintain balance, a pattern that is more energy-consuming and may affect movement coordination (Da Fonseca et al., 2006).

Amateur athletes exhibited a unique sixth synergy, with an activation pattern primarily during the flight phase. This suggests that when coaching amateur athletes, attention should be paid not only to take-off power but also to strengthening their core control capabilities and body posture awareness in the air to reduce unnecessary compensatory muscle activity, which could potentially improve rotational efficiency and landing stability.

Therefore, from a neuromuscular control perspective, the elite Axel jump is the result of a comprehensive neuromuscular optimization. This is reflected not only in the more streamlined and efficient synergistic control that we observed—characterized by more functional muscle weightings and precise activation timing—but likely also in a superior ability for coordinated muscle deactivation and a more efficient utilization of individual muscle capacities for explosive force generation and control.

### 4.3 Study limitations

The limitations of this study include several key points: (1) Study Design: The cross-sectional design used in this study reveals differences between groups but cannot elucidate the longitudinal progression of skill development. (2) Sample: The study participants were exclusively male athletes; therefore, conclusions should be generalized to female athletes with caution. (3) Methodology: Our muscle selection was limited to the lower limbs, excluding core and upper-body musculature. Furthermore, the analysis is subject to the inherent assumptions of the OpenSim model, and the determination of the number of NMF synergies retains a degree of subjectivity despite the objective criteria applied. (4) Technology and Application: The complexity of laboratory-based equipment (OMC/EMG) limits its application in routine training, and future research could explore more accessible technologies like IMUs. Additionally, differences may still exist between the experimental environment and an actual competition setting.

## 5 Conclusion

The following conclusions can be drawn from the above: The dynamic differences in joint angles between amateur and elite athletes during the Axel jump are mainly found in the gliding and take-off phases. These differences are primarily focused on the multi-joint coordination control of the left lower limb, including the adjustment of the hip joint during the gliding phase and the fine-tuning and control strategies of the multi-joint angles of the left lower limb during the take-off phase. The advantage of elite athletes in the Axel jump lies in their highly coordinated performance throughout the entire movement, with more refined and efficient movement strategies. At the neuromuscular control level, elite athletes demonstrate more streamlined and efficient synergistic control. Among the shared synergies between the two groups, elite athletes activate the contribution weights of key muscles earlier, more concentratedly, and more precisely, thereby showing significant advantages in athletic performance.

## Data Availability

The raw data supporting the conclusions of this article will be made available by the authors, without undue reservation.
